# A Pharmacogenetic Approach to Identify Mutant Forms of α-Galactosidase A that Respond to a Pharmacological Chaperone for Fabry Disease

**DOI:** 10.1002/humu.21530

**Published:** 2011-05-19

**Authors:** Xiaoyang Wu, Evan Katz, Maria Cecilia Della Valle, Kirsten Mascioli, John J Flanagan, Jeffrey P Castelli, Raphael Schiffmann, Pol Boudes, David J Lockhart, Kenneth J Valenzano, Elfrida R Benjamin

**Affiliations:** 1 Amicus TherapeuticsCranbury, New Jersey; 2 Institute of Metabolic Disease, Baylor Research InstituteDallas, Texas

**Keywords:** AT1001, Fabry, pharmacological chaperone, α-Galactosidase A

## Abstract

Fabry disease is caused by mutations in the gene (*GLA)* that encodes α-galactosidase A (α-Gal A). The iminosugar AT1001 (GR181413A, migalastat hydrochloride, 1-deoxygalactonojirimycin) is a pharmacological chaperone that selectively binds and stabilizes α-Gal A, increasing total cellular levels and activity for some mutant forms (defined as “responsive”). In this study, we developed a cell-based assay in cultured HEK-293 cells to identify mutant forms of α-Gal A that are responsive to AT1001. Concentration-dependent increases in α-Gal A activity in response to AT1001 were shown for 49 (60%) of 81 mutant forms. The responses of α-Gal A mutant forms were generally consistent with the responses observed in male Fabry patient-derived lymphoblasts. Importantly, the HEK-293 cell responses of 19 α-Gal A mutant forms to a clinically achievable concentration of AT1001 (10 µM) were generally consistent with observed increases in α-Gal A activity in peripheral blood mononuclear cells from male Fabry patients orally administered AT1001 during Phase 2 clinical studies. This indicates that the cell-based responses can identify mutant forms of α-Gal A that are likely to respond to AT1001 in vivo. Thus, the HEK-293 cell-based assay may be a useful aid in the identification of Fabry patients with AT1001-responsive mutant forms. Hum Mutat 32:1–13, 2011. © 2011 Wiley-Liss, Inc.

## Introduction

Fabry disease (MIM♯ 301500) is an X-linked lysosomal storage disorder caused by mutations in the gene (*GLA*;MIM♯ 300644, RefSeq NM_000169.2) that encodes the lysosomal enzyme α-galactosidase A (α-Gal A; EC 3.2.1.22) [Desnick et al., 2001]. α-Gal A is responsible for the first step in the catabolism of neutral glycosphingolipids with terminal α-*D*-galactosyl residues, primarily globotriaosylceramide (GL-3, also known as Gb3 or CTH) [Desnick et al., 2001]. Reduced α-Gal A enzyme activity results in the accumulation of GL-3 in cells and organs throughout the body, which is believed to be a primary contributor to the life-threatening manifestations of Fabry disease, including kidney failure, heart disease, and stroke [Desnick et al., 2001].

The clinical manifestations of Fabry disease span a broad spectrum of severity and generally correlate with a patient's residual α-Gal A levels [Branton et al., 2002; Desnick et al., 2001]. Many of the currently diagnosed patients are males commonly referred to as “classic” Fabry patients [Desnick et al., 2001], who have little or no detectable α-Gal A activity and who are most severely affected. These patients usually experience disease symptoms in adolescence, including acroparaesthesias in the extremities, angiokeratoma, and hypohydrosis. Female patients may be mildly symptomatic, or as severely affected as “classic” males. Many individuals with Fabry disease present with a “later-onset” form, and typically have higher residual α-Gal A levels than “classic” patients. Recent studies suggest that there is a large undiagnosed population of “later-onset” Fabry patients based on systematic screening in hemodialysis, cardiac, and stroke clinics [Bekri et al., 2005; Chimenti et al., 2004; Nakao et al., 1995, 2003; Rolfs et al., 2005; Sachdev et al., 2002; Spada et al., 2006].

The iminosugar 1-deoxygalactonojirimycin (DGJ, also referred to as migalastat) is an analog of the terminal galactose of GL-3 that can selectively and reversibly bind to the active site of wild-type and mutant forms of α-Gal A [Fan et al., 1999a]. Binding of DGJ stabilizes α-Gal A, thereby increasing the ability of newly synthesized enzyme to pass the quality control system of the endoplasmic reticulum (ER), traffic to lysosomes [Fan et al., 1999a; Fan and Ishii, 2003; Yam et al., 2005, 2006], and reduce the storage of lysosomal GL-3 both in cultured cells and in vivo [Fan et al., 1999a; Ishii et al., 2009; Khanna et al., 2010; Yam et al., 2005, 2006]. As such, DGJ acts as a “pharmacological chaperone” of α-Gal A. It has been hypothesized that a pharmacological chaperone may be a viable treatment for Fabry disease, serving as an alternative to enzyme replacement therapy for some patients [Fan et al., 1999a; Fan and Ishii, 2003; Yam et al., 2005, 2006]. Accordingly, DGJ is the active component of an investigational new drug, migalastat hydrochloride (AT1001, GR181413A), that currently is in clinical development to evaluate its safety and efficacy as a potential treatment for Fabry disease [Schiffmann et al., 2008].

Mutant forms that are most likely to show increased total cellular α-Gal A activity in Fabry patients treated with AT1001 are physically unstable, prone to inefficient or aberrant folding, have deficient lysosomal trafficking, and/or show increased levels in cultured cells upon binding and stabilization by AT1001. Such mutant forms are defined as “responsive” [Bernier et al., 2004; Desnick, 2004; Desnick et al., 2001; Garman and Garboczi, 2002, 2004; Ioannou et al., 1998; Ishii et al., 1993, 1996]. Missense mutations in *GLA* often lead to the expression of responsive mutant forms of α-Gal A [Benjamin et al., 2009; Fan et al., 1999a; Ishii et al., 1996, 2007; Lemansky et al., 1987; Shin et al., 2008; Yam et al., 2005, 2006]. In addition, some mutant forms of α-Gal A arising from either in-frame insertions, deletions, or multiple-site missense mutations of *GLA*, which affect only a small number of amino acids (Human Gene Mutation Database; http://www.hgmd.cf.ac.uk/ac/index.php) and retain sufficient α-Gal A structure and function, may also be responsive to AT1001 [Benjamin et al., 2009; Desnick, 2004; Desnick et al., 2001; Garman and Garboczi, 2002, 2004]. In contrast, mutations in *GLA* that impair the synthesis of α-Gal A, lead to the absence of full-length protein (e.g., frame-shift, nonsense, larger deletion mutations, and large insertions), or that significantly affect substrate binding or catalytic activity are not expected to result in mutant forms that can be stabilized by AT1001 [Desnick, 2004; Desnick et al., 2001; Garman and Garboczi, 2002, 2004; Lai et al., 2003]. Mutant forms that are not affected by AT1001 are defined as “non-responsive.”

Previous studies have shown that AT1001 increases the cellular levels and activity of some missense mutant forms of α-Gal A in cultured cell lines (e.g., lymphoblasts or fibroblasts) derived from Fabry patients [Benjamin et al., 2009; Fan et al., 1999a; Ishii et al., 2007; Shin et al., 2007]. The AT1001-mediated increases in α-Gal A activity were concentration-dependent, consistent across cell lines and cell types from different patients with the same mutation, and have been demonstrated for mutant forms of the enzyme that are associated with both “classic and “later-onset” Fabry disease [Benjamin et al., 2009; Ishii et al., 2009; Yam et al., 2005, 2006]. As such, it has been speculated that the cultured cell-based responses of different mutant forms of α-Gal A to clinically achievable concentrations of AT1001 may be useful to identify Fabry patients suitable for treatment with AT1001 [Benjamin et al., 2009; Shin et al., 2007, 2008]. To date, however, an evaluation of whether the mutant forms of α-Gal A that are responsive to AT1001 in cultured cells can also respond in Fabry patients has not been reported. Thus, we have developed a cell-based assay in HEK-293 cells that can identify mutant forms of α-Gal A that are responsive to AT1001. Importantly, this assay does not rely on patient-derived cell lines. Furthermore, we determined if the responses measured in this assay can identify mutant forms of α-Gal A that respond to AT1001 in Fabry patient-derived cell lines and in peripheral blood mononuclear cells (PBMCs) of Fabry patients administered the drug.

## Materials and Methods

### Materials

The HEK-293 cell line (GripTite™ 293 MSR) was purchased from Invitrogen (Carlsbad, CA). AT1001 (DGJ hydrochloride; migalastat hydrochloride) was synthesized by Cambridge Major Laboratories (Germantown, WI). The synthetic fluorogenic substrate for α-Gal A, 4-methylumbelliferone-α-D-galactopyranoside (4-MUG), was manufactured in bulk quantity (100 g) by Melford Laboratories Ltd (Suffolk, UK). All other reagents were purchased from Sigma-Aldrich (St. Louis, MO) unless otherwise indicated.

### Mutagenesis

Full-length cDNA encoding wild-type α-Gal A (GLA, RefSeq NM_000169.2) was subcloned into the expression vector pcDNA6 (Invitrogen) from pCNX2-GLA [Yasuda et al., 2004]. All mutations in the *GLA* cDNA were generated by site-directed mutagenesis within pcDNA6 following standard molecular biology protocols. Briefly, specific primer pairs were designed containing the desired mutation. The mutagenesis was performed by polymerase chain reaction (PCR) using *PfuUltra* high-fidelity DNA polymerase (Stratagene, La Jolla, CA) in a thermocycler (MJ Research, Waltham, MA). The reactions contained a total volume of 50 µl with the following: 41.5 µl dH_2_O, 5.0 µl 10 × *PfuUltra* HF reaction buffer, 0.5 µl each of Forward (or 5′-) and Reverse (or 3′-) primers (50 µM), 1 µl dNTP mix (containing 10 mM each of dA, dT, dC, dG), 1 µl pcDNA6*-GLA* template (2 ng/µl), 0.5 µl *PfuUltra* HF DNA polymerase. The PCR amplification consisted of 16 reaction cycles (30 sec denaturation at 94°C; 30 sec annealing at 55–60°C depending on the specific melting temperature of a given primer pair; 6 min elongation at 68°C). Afterward, 0.5 µl *Dpn*I (New England Biolabs, Ipswich, MA) was added to each reaction and incubated at 37°C for 2 hr. A volume of 7.5 µl for each mutagenesis reaction was used to transform 50 µl DH5α cells (New England Biolabs). Cells were plated on LB-Agar (Lenox L Agar) (Invitrogen) with 75 µg/ml ampicillin, and incubated at 37°C overnight. Bacterial colonies were picked, grown overnight in liquid Miller's LB broth (ACROS, Geel, Belgium) with ampicillin shaking at 37°C, and plasmid DNA was extracted using the QuickLyse Miniprep Kit (Qiagen, Valencia, CA). Mutants were confirmed by sequencing the full-length *GLA* cDNA insert on the pcDNA vector. Large-scale plasmid preparations were made using the PureYield™ Plasmid Maxiprep Kit (Promega, Madison, WI). Stock DNA was diluted to 100 µg/ml in water and resequenced prior to transfection.

### Transient Transfection and AT1001 Incubation

HEK-293 cells were seeded in sterile clear-bottom 96-well plates (Corning Life Sciences, Lowell, MA) at a density of 7,500–10,000 cells/well and incubated at 37°C, 5% CO_2_ for 24 hr. For transfection, pcDNA6 plasmid with a mutated *GLA* insert was diluted in fresh serum-free Opti-MEM (Invitrogen) and incubated with fresh transfection HD reagent at a ratio of 1:3.5 (plasmid DNA:HD reagent) (Invitrogen) for 5 min at room temperature in a total volume of 300 µl. Afterward, 5 µl of the freshly prepared transfection mix (containing 0.1 µg of plasmid DNA) were added to each of 48 wells in the 96-well plate (two mutants/plate). Cells were incubated at 37°C, 5% CO_2_ for 1 hr and then vehicle control (5.5 µl media) or 20-fold concentrated stock 1:3 serial dilutions of AT1001 were added in quadruplicate wells for each transfected mutant form. The initial concentration range of AT1001 was 50 nM to 1 mM; if the concentration–response curve did not reach saturation within this range, the concentration range was adjusted to include AT1001 concentrations as high as 20 mM for subsequent determinations. Cells were then incubated for 4 to 5 days before lysis and assay. AT1001 concentration–response curves in wild-type and R301Q transfected cells were run in parallel as transfection and positive response controls, respectively, in every assay. Parallel transfection of wild-type *GLA* was considered to be the most appropriate transfection control for missense and small deletion or insertion *GLA* mutants, as the cDNA sequence differs only by one or a few base pairs, and it is the most commonly used transfection control for transiently expressed mutant α-Gal A assays [Fan and Ishii, 2003; Ishii et al., 2007]. In addition, quantitative real-time PCR measurement of the amount of plasmid DNA recovered from HEK-293 cells 5 days after transfection was done for wild-type and several *GLA* mutants. These measurements detected *GLA* cDNA-specific signals that were greater than 10-fold above background (i.e., pcDNA transfected or untransfected cells), indicating that the transfection efficiencies across different independent assays and mutations were generally very good regardless of baseline activity, protein level, or response to AT1001 (data not shown). R301Q is an appropriate positive control as its responsiveness to AT1001 has been well established in cultured cells and in vivo [Asano et al., 2000; Benjamin et al., 2009; Fan et al., 1999a; Fan and Ishii, 2003; Ishii et al., 2007, 2009; Shin et al., 2007; Yam et al., 2005, 2006].

### **α**-Gal A Enzyme Activity Measurement

HEK-293 cells were washed twice with phosphate-buffered saline (PBS), incubated in 200 µl fresh media at 37°C, 5% CO_2_ for 2 hr, and washed twice with PBS. Afterward, cells were lysed in 60 µl Lysis Buffer (27 mM sodium citrate, 46 mM sodium phosphate dibasic, 0.5% Triton X-100, pH 4.6). Lysates (10 µl) were added to 50 µl Assay Buffer (Lysis Buffer without Triton X-100), containing 6 mM 4-MUG and 117 mM *N*-acetyl-D-galactosamine (GalNac, an inhibitor of α-*N*-acetylgalactosaminidase, a lysosomal enzyme present in the cell lysates that has been shown to hydrolyze 4-MUG [Mayes et al., 1981]), and incubated at 37°C for 1 hr. Stop Solution (0.4 M glycine, pH 10.8; 70 µl) was then added and fluorescence read on a Victor plate reader (Perkin-Elmer, Waltham, MA) at 355 nm excitation and 460 nm emission. To correct for endogenous α-Gal A activity in HEK-293 cells, AT1001 concentration–response curves in pcDNA vector only-transfected cells were run in parallel and raw fluorescence counts in lysates from these cells were subtracted from those in mutant- or wild-type-transfected cells. The resultant specific raw fluorescence counts for each individual form of α-Gal A as a function of AT1001 concentration was determined. A Micro BCA Protein Assay Kit (Pierce, Rockford, IL) was used according to the manufacturer's instructions to determine protein concentration from 40 µl of cell lysate from each well. The total protein amount in each well was used to normalize enzyme activity. A 4-methylumbelliferone (4-MU) standard curve ranging from 1.3 nM to 30 µM was run in parallel for calculation of absolute α-Gal A activity expressed as nmol of 4-MU released/mg protein/hr (nmol/mg protein/hr) and further normalized to percentage of untreated wild-type (% WT) enzyme activity. At least three independent experiments were carried out for each mutation. A statistically significant increase in α-Gal A levels in response to AT1001 was determined by a two-tailed, paired *t*-test (*P*<0.05) comparing the enzyme activity in the absence of AT1001 to the maximally achieved enzyme activity in the presence of AT1001 for each mutant form of α-Gal A.

### **α**-Gal A Western Blot

Transfected HEK-293 cells were incubated in the absence or presence of 0.1 and 1 mM AT1001 for 4 to 5 days. After removing media and washing twice with 200 µl PBS, 40 µl Lysis Buffer containing protease inhibitors (Roche) were added to each well of the 96-well plate and the plate was shaken at room temperature for 15 min to lyse the cells. The total protein concentration in each well was measured from 10 µl of lysate using the MicroBCA Protein Assay Kit. Remaining lysates were stored at −80°C for Western blot analysis. Briefly, 1 µg of total protein was loaded on a 4–12% NuPAGE gradient gel (Invitrogen) and electrophoresed at 150 volts for 90 min. Proteins on the gel were then transferred to nitrocellulose membranes using the iBlot™ Dry Blotting System (Invitrogen). Immunoblots were probed with a 1:500 dilution of rabbit antihuman GAPDH polyclonal antibody (Abcam Inc., Cambridge, MA) and a 1:1,250 dilution of rabbit antihuman α-Gal A polyclonal antibody (SH-006-CR0020-08; provided by Shire Human Genetic Therapies, Inc., Cambridge, MA) overnight on a platform shaker. After washing, blots were incubated for 45 min with a 1:5,000 dilution of horseradish peroxidase-conjugated goat antirabbit secondary antibody (Pierce), and were then developed with enhanced chemiluminescence reagents (Pierce) and imaged on a Kodak Image Station 4000R (Carestream Molecular Imaging, Rochester, NY).

### Locations of Point Mutations in **α**-Gal A

The locations of the amino acid residues that have been altered by site-directed mutagenesis and tested in the HEK-293 cell-based assay were mapped onto the previously determined structure of human α-Gal A (PDB:1R47) [Garman and Garboczi, 2004]. The illustrations of the three-dimensional structures were generated with SWISS-PDB viewer [Guex and Peitsch, 1997] and rendered with Persistence of Vision Raytracer (Version 3.6).

### Phase 2 Clinical Studies with AT1001 in Fabry Patients

Three Phase 2 clinical trials with AT1001 were conducted in male patients with Fabry disease (see ClinicalTrials.gov; NCT00214500, NCT00283959, and NCT00283933). Institutional review board approval was obtained for all centers involved in the studies and all patients gave written informed consent to participate.

### α-Gal A Assay in PBMCs

α-Gal A levels in PBMCs were measured in Fabry patients from the above trials to assess their response to AT1001. Blood was drawn into an 8-ml Vacutainer® CPT™ tube (Becton Dickinson, Franklin Lakes, NJ) from each patient just prior to the first administration of AT1001, and at various timepoints afterward in accordance with the different clinical protocols. PBMCs were immediately harvested according to the manufacturer's protocol, pelleted, and frozen at −80°C. Cell pellets were thawed on ice and lysed by sonication in PBMC Lysis Buffer (2.7 mM citric acid, 4.6 mM sodium phosphate dibasic, pH 5.5). Lysates were frozen on a dry ice/ethanol bath and stored at −80°C. To assay, lysates (50 µl) were combined with 50 µl 117 mM GalNac and 50 µl 5 mM 4-MUG (each dissolved in 27 mM citric acid, 46 mM sodium phosphate dibasic, pH 4.6; 4-MUG is dissolved in DMSO first in a ratio of 30 µmol/ml). Reaction mixtures were incubated at 37°C for 1 hr. Afterward, 100 µl Stop Solution (0.2 M NaOH-glycine, pH 10.7) was added, and fluorescence was read on a SPECTRAmax® Gemini XS Spectrophotometer (Molecular Devices, Downingtown, PA) at 375 nm excitation and 450 nm emission. A 4-MU standard curve ranging from 50 nM to 20 mM was run in parallel. The BCA Protein Assay Kit was used to determine the protein concentration from 25 µl of cell lysate. Absolute α-Gal A activity was expressed as nmol/mg protein/hr and further normalized to percentage of normal enzyme activity.

### Data Analysis

The maximum α-Gal A activity was obtained from the top of the sigmoidal concentration–response curve after nonlinear regression analysis in GraphPad Prism, version 4.02 (San Diego, CA). The baseline α-Gal A activity was determined in the absence of AT1001. The α-Gal A activity limit of quantification (LOQ) in the HEK-293 cell assay was defined as 300 nmol/mg protein/hr (this value is ≥1.645*standard deviation of the α-Gal A activity in pcDNA vector-only transfected cells in ≥90% of the experiments). Thus, any measured or calculated value of mutant or wild-type α-Gal A activity in the HEK-293 cell-based assay that was below this LOQ was imputed as a value of zero. The maximum relative increase was calculated from the maximum α-Gal A activity in cell lysates after AT1001 incubation divided by the baseline α-Gal A activity (not calculable for mutant forms with a baseline activity value of zero). The concentration of AT1001 that resulted in 50% of the maximal increase in α-Gal A activity (EC_50_ value) was calculated from the sigmoidal concentration–response curve by the GraphPad Prism software. The α-Gal A activity at the clinically achievable concentration of 10 µM AT1001 [Schiffmann et al., 2008] was calculated using Equation 1 in Microsoft Office Excel 2003 or 2007 (Redmond, WA). Analyses to determine p-values for statistical significance were carried out using Microsoft Office Excel 2003 or 2007. Analyses to determine the Pearson correlation coefficient and two-tailed correlation p-value were carried out using GraphPad Prism, version 4.02.

Equation 1. α-Gal A activity at 10 µM AT1001:
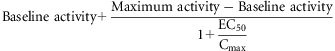
where EC_50_ is the concentration of AT1001 that yields 50% of the maximal increase in α-Gal A activity and C_max_ is 10 µM AT1001. C_max_ refers to the approximate average maximum concentration of AT1001 measured in plasma of Fabry patients following a single oral administration of 150 mg AT1001 [Schiffmann et al., 2008].

### Mutation Nomenclature

Nucleotide numbering reflects cDNA numbering with +1 corresponding to the A of the ATG translation initiation codon in the reference sequence (*GLA*, RefSeq NM_000169.2). The initiation codon is codon 1.

## Results

### Identification of Mutant Forms of α-Gal A That are Responsive to AT1001

**Figure 1:**
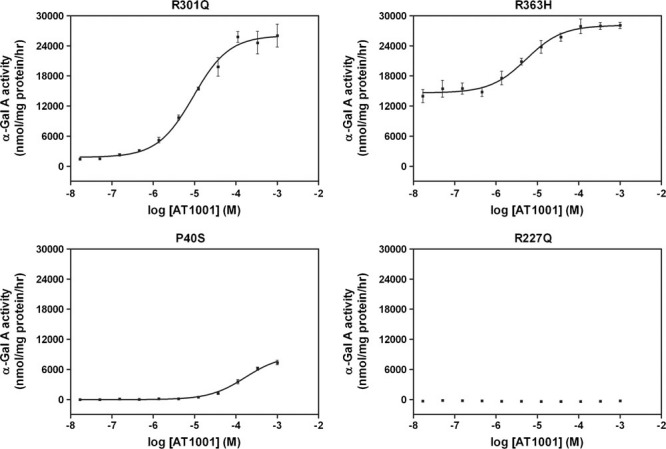
Responses to AT1001 for different mutant forms of α-Gal A. Representative α-Gal A activity (expressed as nmol 4-MU released/mg protein/hr) in lysates from HEK-293 cells transiently transfected with the indicated mutant α-Gal A construct and incubated with increasing concentrations of AT1001 is shown. AT1001 increased the activity of R301Q (top left panel), R363H (top right panel), and P40S (bottom left panel), but had no effect on R227Q (bottom right panel). Data points are the mean ± SEM of quadruplicate determinations. In the experiments shown, α-Gal A activity was increased 14.6-fold and 1.9-fold for R301Q and R363H, respectively (relative increase was not calculated for P40S because its baseline is 0). The EC_50_ values were 9.3, 5.4, and 170 µM for R301Q, R363H, and P40S, respectively. The data shown are representative of 24 (R301Q), 4 (R363H), 4 (P40S), and 3 (R227Q) independent experiments.

Eighty-one Fabry disease-causing missense mutations (including two double mutations) were identified in male Fabry patient-derived lymphoblasts or subjects enrolled in the Phase 2 clinical trials for AT1001. Each mutation was then introduced individually by site-directed mutagenesis into a human α-Gal A cDNA residing in a mammalian expression vector to create the recombinant expression construct that encoded each of the different mutant forms of α-Gal A. After transient transfection, HEK-293 cells were incubated without and with increasing concentrations of AT1001 for 4 to 5 days. The AT1001 potency (EC_50_ value) and maximal increase in α-Gal A activity after AT1001 incubation were determined for each transiently expressed mutant form (Fig. 1).

**Figure 2:**
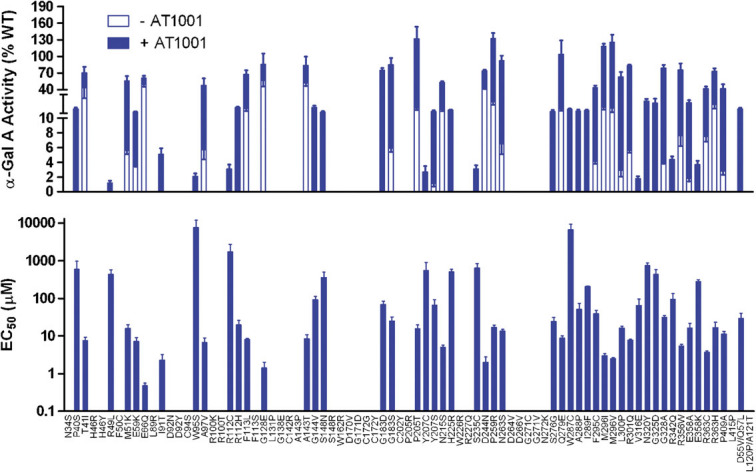
AT1001 increases the activity of different α-Gal A mutant forms with varying magnitude and potency. Top panel: Eighty-one mutant forms were evaluated in the HEK-293 cell-based assay for response to incubation with AT1001. The average baseline (open bars) and maximally increased (blue/dark bars) α-Gal A activity in the absence or presence of AT1001, respectively, are shown. The data have been normalized to the α-Gal A activity of untreated wild-type. Bottom panel: The average EC_50_ values (expressed as µM) for AT1001 to increase the α-Gal A activity of each mutant form are shown. For both panels, α-Gal A mutant forms were plotted in order of their positions on the amino acid sequence. Bars represent mean ± SEM of at least three independent experiments conducted for each mutant form. Mutant forms with no associated bar did not have any quantifiable baseline α-Gal A activity nor response to AT1001 at the concentrations tested. [Color figures can be viewed in the online issue, which is available at http://www.wiley.com/humanmutation.]

Overall, 49 of the 81 mutant forms (60%) showed a concentration-dependent and statistically significant increase in α-Gal A activity in response to AT1001 in HEK-293 cells (Table 1 and Fig. 2). Baseline α-Gal A activity ranged from 0 to 57% of wild-type. The maximum α-Gal A activity obtained after incubation with AT1001 ranged from 1.2 to 132% of wild type. The maximum relative increase in α-Gal A activity ranged from 1.3-fold for E66Q (c.196G>C) to greater than 20-fold for L300P (c.899T>C) and G328A (c.983G>C) (mutant forms with zero α-Gal A activity at baseline were excluded from this analysis). The EC_50_ values ranged from 0.5 µM for E66Q to greater than 800 µM for W95S (c.284G>C), R112C (c.334C>T), and W287C (c.861G>C).

**Table 1:** AT1001-Mediated Responses to AT1001 of Mutant Forms of α-Gal A Expressed in HEK-293 Cells

		−AT1001		+AT1001				
					
Protein change	cDNA change	α-Gal A Activity	% WT	α-Gal A Activity	% WT	Relative increase	EC_50_ (µM)	*n*
Wild-type	Wild-type	33,217 ± 1,077	100.0 ± 0.0	38,813 ± 1,553	117 ± 3	–	–	24
p.N34S	c.101A>G	0.0 ± 0.0	0.0 ± 0.0	0.0 ± 0.0	0.0 ± 0.0	–	–	8
p.P40S	c.118C>T	0.0 ± 0.0	0.0 ± 0.0	6,462 ± 940	16.7 ± 2.7	NC	592 ± 377	4
p.T41I	c.122C>T	18,436 ± 5,427	43.1 ± 9.0	29,293 ± 3,745*	70.4 ± 11.1	1.7 ± 0.2	7.6 ± 1.7	4
p.H46R	c.137A>G	0.0 ± 0.0	0.0 ± 0.0	0.0 ± 0.0	0.0 ± 0.0	–	–	3
p.H46Y	c.136C>T	0.0 ± 0.0	0.0 ± 0.0	0.0 ± 0.0	0.0 ± 0.0	–	–	3
p.R49L	c.146G>T	0.0 ± 0.0	0.0 ± 0.0	370 ± 81*	1.2 ± 0.3	NC	433 ± 138	3
p.F50C	c.149T>G	0.0 ± 0.0	0.0 ± 0.0	0.0 ± 0.0	0.0 ± 0.0	–	–	3
p.M51K	c.152T>A	1,857 ± 358	5.6 ± 0.5	18,557 ± 3,974*	56.1 ± 8.6	9.2 ± 1.8	15.9 ± 4.2	3
p.E59K	c.175G>A	1,108 ± 40	3.6 ± 0.2	3,742 ± 180**	12.2 ± 0.7	3.9 ± 0.2	7.2 ± 1.9	3
p.E66Q	c.196G>C	14,739 ± 710	49.1 ± 3.8	18,388 ± 722**	61.2 ± 3.8	1.3 ± 0.0	0.5 ± 0.1	5
p.L89R	c.266T>G	0.0 ± 0.0	0.0 ± 0.0	0.0 ± 0.0	0.0 ± 0.0	–	–	3
p.I91T	c.272T>C	0.0 ± 0.0	0.0 ± 0.0	1,556 ± 162*	5.1 ± 0.8	NC	2.3 ± 0.9	3
p.D92N	c.274G>A	0.0 ± 0.0	0.0 ± 0.0	0.0 ± 0.0	0.0 ± 0.0	–	–	3
p.D92Y	c.274G>T	0.0 ± 0.0	0.0 ± 0.0	0.0 ± 0.0	0.0 ± 0.0	–	–	3
p.C94S	c.281G>C	0.0 ± 0.0	0.0 ± 0.0	0.0 ± 0.0	0.0 ± 0.0	–	–	9
p.W95S	c.284G>C	0.0 ± 0.0	0.0 ± 0.0	672 ± 112	2.1 ± 0.4	NC	7,560 ± 4,329	6
p.A97V	c.290C>T	1,898 ± 406	5.7 ± 1.3	15,832 ± 3,968*	48.0 ± 12.5	8.3 ± 0.5	6.7 ± 2.2	4
p.R100K	c.299G>A	0.0 ± 0.0	0.0 ± 0.0	0.0 ± 0.0	0.0 ± 0.0	–	–	3
p.R100T	c.299G>C	0.0 ± 0.0	0.0 ± 0.0	0.0 ± 0.0	0.0 ± 0.0	–	–	3
p.R112C	c.334C>T	0.0 ± 0.0	0.0 ± 0.0	1,042 ± 202*	3.1 ± 0.6	NC	1,720 ± 991^a^	4
p.R112H	c.335G>A	0.0 ± 0.0	0.0 ± 0.0	5,676 ± 590**	19.0 ± 1.3	17.1 ± 5.4	19.7 ± 6.2	3
p.F113L	c.337T>C	5,960 ± 1,230	17.3 ± 3.6	23,244 ± 1,348**	67.6 ± 7.4	4.0 ± 0.4	8.0 ± 0.6	3
p.F113S	c.338T>C	0.0 ± 0.0	0.0 ± 0.0	0.0 ± 0.0	0.0 ± 0.0	–	–	3
p.G128E	c.383G>A	10,719 ± 883	56.9 ± 11.2	16,409 ± 1,793**	85.8 ± 19.4	1.5 ± 0.1	1.4 ± 0.6	8
p.L131P	c.392T>C	0.0 ± 0.0	0.0 ± 0.0	0.0 ± 0.0	0.0 ± 0.0	–	–	3
p.G138E	c.413G>A	0.0 ± 0.0	0.0 ± 0.0	0.0 ± 0.0	0.0 ± 0.0	–	–	3
p.C142R	c.424T>C	0.0 ± 0.0	0.0 ± 0.0	0.0 ± 0.0	0.0 ± 0.0	–	–	3
p.A143P	c.427G>C	0.0 ± 0.0	0.0 ± 0.0	0.0 ± 0.0	0.0 ± 0.0	–	–	3
p.A143T	c.427G>A	15,499 ± 2,674	51.9 ± 5.2	23,131 ± 3,312***	83.8 ± 16.0	1.5 ± 0.1	8.4 ± 2.3	6
p.G144V	c.431G>T	0.0 ± 0.0	0.0 ± 0.0	5,932 ± 890**	19.5 ± 2.6	NC	93.4 ± 19.0	4
p.S148N	c.443G>A	0.0 ± 0.0	0.0 ± 0.0	4,003 ± 693*	11.9 ± 1.3	NC	360 ± 137	3
p.S148R	c.444T>G	0.0 ± 0.0	0.0 ± 0.0	0.0 ± 0.0	0.0 ± 0.0	–	–	3
p.W162R	c.484T>C	0.0 ± 0.0	0.0 ± 0.0	0.0 ± 0.0	0.0 ± 0.0	–	–	3
p.D170V	c.509A>T	0.0 ± 0.0	0.0 ± 0.0	0.0 ± 0.0	0.0 ± 0.0	–	–	3
p.G171D	c.512G>A	0.0 ± 0.0	0.0 ± 0.0	0.0 ± 0.0	0.0 ± 0.0	–	–	4
p.C172G	c.514T>G	0.0 ± 0.0	0.0 ± 0.0	0.0 ± 0.0	0.0 ± 0.0	–	–	3
p.C172Y	c.515G>A	0.0 ± 0.0	0.0 ± 0.0	0.0 ± 0.0	0.0 ± 0.0	–	–	4
p.G183D	c.548G>A	0.0 ± 0.0	0.0 ± 0.0	22385 ± 1,897**	75.0 ± 4.2	NC	68.8 ± 16.5	4
p.G183S	c.547G>A	1,888 ± 116	5.9 ± 0.5	27,614 ± 4,530*	84.9 ± 12.5	14.3 ± 1.1	25.2 ± 6.9	3
p.C202Y	c.605G>A	0.0 ± 0.0	0.0 ± 0.0	0.0 ± 0.0	0.0 ± 0.0	–	–	6
p.P205R	c.614C>G	0.0 ± 0.0	0.0 ± 0.0	0.0 ± 0.0	0.0 ± 0.0	–	–	9
p.P205T	c.613C>A	5,524 ± 747	18.2 ± 2.8	40,178 ± 6,172*	132 ± 21	7.6 ± 1.5	15.6 ± 4.3	4
p.Y207C	c.620A>G	0.0 ± 0.0	0.0 ± 0.0	848 ± 176*	2.7 ± 0.8	NC	547 ± 351	3
p.Y207S	c.620A>C	364 ± 100	1.1 ± 0.4	4,195 ± 460***	12.8 ± 1.9	13.5 ± 2.4	66.4 ± 26.6	5
p.N215S	c.644A>G	4,898 ± 814	15.7 ± 2.4	16,400 ± 395***	53.2 ± 2.4	3.5 ± 0.5	5.0 ± 0.7	3
p.H225R	c.674A>G	0.0 ± 0.0	0.0 ± 0.0	4,350 ± 366***	15.1 ± 1.0	NC	502 ± 87	3
p.W226R	c.676T>C	0.0 ± 0.0	0.0 ± 0.0	0.0 ± 0.0	0.0 ± 0.0	–	–	4
p.R227Q	c.680G>A	0.0 ± 0.0	0.0 ± 0.0	0.0 ± 0.0	0.0 ± 0.0	–	–	4
p.S235C	c.704C>G	0.0 ± 0.0	0.0 ± 0.0	1,028 ± 146*	3.1 ± 0.5	NC	628 ± 211	5
p.D244N	c.730G>A	13,394 ± 820	43.2 ± 1.5	22,948 ± 1,763**	73.9 ± 2.2	1.7 ± 0.0	2.0 ± 0.8	3
p.P259R	c.776C>G	8,585 ± 1131	28.1 ± 4.6	40,724 ± 1,869**	132 ± 10	4.9 ± 0.5	16.8 ± 2.7	3
p.N263S	c.788A>G	1,928 ± 436	6.5 ± 1.4	27,458 ± 3,232**	92.3 ± 9.1	14.9 ± 2.2	13.5 ± 1.6	3
p.D264V	c.791A>T	0.0 ± 0.0	0.0 ± 0.0	0.0 ± 0.0	0.0 ± 0.0	–	–	3
p.D266V	c.797A>T	0.0 ± 0.0	0.0 ± 0.0	0.0 ± 0.0	0.0 ± 0.0	–	–	3
p.G271C	c.811G>T	0.0 ± 0.0	0.0 ± 0.0	0.0 ± 0.0	0.0 ± 0.0	–	–	4
p.G271V	c.812G>T	0.0 ± 0.0	0.0 ± 0.0	0.0 ± 0.0	0.0 ± 0.0	–	–	5
p.N272K	c.816C>A	0.0 ± 0.0	0.0 ± 0.0	0.0 ± 0.0	0.0 ± 0.0	–	–	3
p.S276G	c.826A>G	0.0 ± 0.0	0.0 ± 0.0	4,492 ± 41	12.7 ± 2.5	NC	24.3 ± 6.8	7
p.Q279E	c.835C>G	5,138 ± 854	16.6 ± 2.5	32,048 ± 8,092*	104 ± 25	6.1 ± 0.9	8.8 ± 1.3	4
p.W287C	c.861G>C	0.0 ± 0.0	0.0 ± 0.0	5,809 ± 973*	16.4 ± 1.1	NC	6,727 ± 2,634^a^	3
p.A288P	c.862G>C	0.0 ± 0.0	0.0 ± 0.0	4,856 ± 650*	13.6 ± 2.3	NC	51 ± 23	3
p.I289F	c.865A>T	0.0 ± 0.0	0.0 ± 0.0	4,900 ± 90**	14.3 ± 1.4	NC	209 ± 5	3
p.F295C	c.884T>G	1,257 ± 171	4.1 ± 0.3	13,482 ± 982**	44.0 ± 3.3	12.0 ± 1.4	38.8 ± 9.5	3
p.M296I	c.888G>A	5,909 ± 802	19.5 ± 3.5	36,470 ± 1,157**	118 ± 5	6.2 ± 1.1	3.0 ± 0.4	3
p.M296V	c.886A>G	5,575 ± 1837	18.3 ± 6.5	38,397 ± 1,949**	126 ± 14	7.6 ± 1.9	2.5 ± 0.2	3
p.L300P	c.899T>C	864 ± 260	2.9 ± 0.9	18,584 ± 2,654*	62.9 ± 9.0	24.5 ± 3.4	16.4 ± 1.8	3
p.R301Q	c.902G>A	1,854 ± 117	5.6 ± 0.3	27,765 ± 1,060***	83.7 ± 1.9	16.0 ± 1.0	7.7 ± 0.4	24
p.V316E	c.947T>A	0.0 ± 0.0	0.0 ± 0.0	542 ± 99***	1.8 ± 0.3	NC	64.4 ± 31.2	7
p.N320Y	c.958A>T	0.0 ± 0.0	0.0 ± 0.0	10,181 ± 631**	29.6 ± 3.5	NC	738 ± 133^a^	3
p.G325D	c.974G>A	0.0 ± 0.0	0.0 ± 0.0	8,439 ± 1,441***	26.2 ± 7.2	NC	430 ± 144^a^	3
p.G328A	c.983G>C	1,320 ± 136	3.9 ± 0.1	26,534 ± 718***	79.0 ± 5.7	21.0 ± 1.9	31.3 ± 3.8	3
p.R342Q	c.1025G>A	0.0 ± 0.0	0.0 ± 0.0	1,175 ± 137*	4.4 ± 0.4	NC	95.0 ± 39.3	4
p.R356W	c.1066C>T	2,352 ± 276	7.6 ± 1.4	23,720 ± 2,474*	75.6 ± 11.6	10.1 ± 1.3	5.4 ± 0.6	3
p.E358A	c.1073A>C	639 ± 200	1.8 ± 0.4	8,680 ± 902**	26.9 ± 4.2	16.0 ± 4.4	16.4 ± 5.4	4
p.E358K	c.1072G>A	0.0 ± 0.0	0.0 ± 0.0	1,417 ± 136*	3.7 ± 0.5	NC	281 ± 32	4
p.R363C	c.1087C>T	2,385 ± 119	7.5 ± 0.7	13,603 ± 1,559*	42.0 ± 3.9	5.5 ± 0.4	3.7 ± 0.3	3
p.R363H	c.1088G>A	8,302 ± 2,086	24.1 ± 6.8	25,721 ± 2,292**	73.4 ± 5.3	3.3 ± 0.5	16.7 ± 6.5	4
p.P409A	c.1225C>G	834 ± 116	2.8 ± 0.5	12,572 ± 2,254*	42.0 ± 7.8	15.3 ± 1.6	11.3 ± 1.9	3
p.L415P	c.1244T>C	0.0 ± 0.0	0.0 ± 0.0	0.0 ± 0.0	0.0 ± 0.0	–	–	7
p.D55V/Q57L	c.164A>T/170C>T	0.0 ± 0.0	0.0 ± 0.0	5,557 ± 800*	16.9 ± 1.9	NC	29.4 ± 10.8	4
p.L120P/A121T	c.359T>C/361G>A	0.0 ± 0.0	0.0 ± 0.0	0.0 ± 0.0	0.0 ± 0.0	–	–	4

Nucleotide numbering reflects cDNA numbering with +1 corresponding to the A of the ATG translation initiation codon in the reference sequence (*GLA*, RefSeq NM_000169.2). The initiation codon is codon 1. The α-Gal A activity (+AT1001) is the maximum α-Gal A activity determined as described in Materials and Methods. For mutant forms with significant increases, a corresponding EC_50_ value for AT1001 was calculated. For mutant forms with EC_50_ values > 800 µM, the maximum α-Gal A activity values (+AT1001) are those measured at the highest tested concentrations of AT1001; these EC_50_ values are extrapolated because of incomplete saturation at the tops of the concentration–response curves.

a The top of the AT1001 concentration–response curve was 10 mM. Data are expressed as the mean ± SEM of “n” independent experiments. Activity below the LOQ is indicated as 0.0 ± 0.0. NC refers to “not calculable.” — indicates no significant increase in α-Gal A activity seen.

* Indicates *P*<0.05

** indicates *P*<0.01

*** indicates *P*<0.001 for enzyme activity (nmol of free 4-MU released/mg protein/hr) measured in lysates of cells incubated in the presence of AT1001 compared to the baseline activity.

Significance was determined by two-tailed, paired *t*-test.

Interestingly, 22 of the 54 mutant forms with baseline activity below the limit of quantification were responsive, whereas 32 showed no response to AT1001 at any concentration tested (Table 1). In contrast, all of 27 mutant forms with quantifiable baseline α-Gal A activity were responsive to AT1001. However, wild-type α-Gal A transiently expressed in HEK-293 cells did not consistently show a concentration-dependent increase in activity after incubation with AT1001 (Table 1, also see Discussion). Sixteen of the mutant forms that were responsive had AT1001 EC_50_ values of less than or equal to 10 µM (Table 1 and Fig. 2).

### AT1001 Increases Mutant α-Gal A Protein Levels

**Figure 3:**
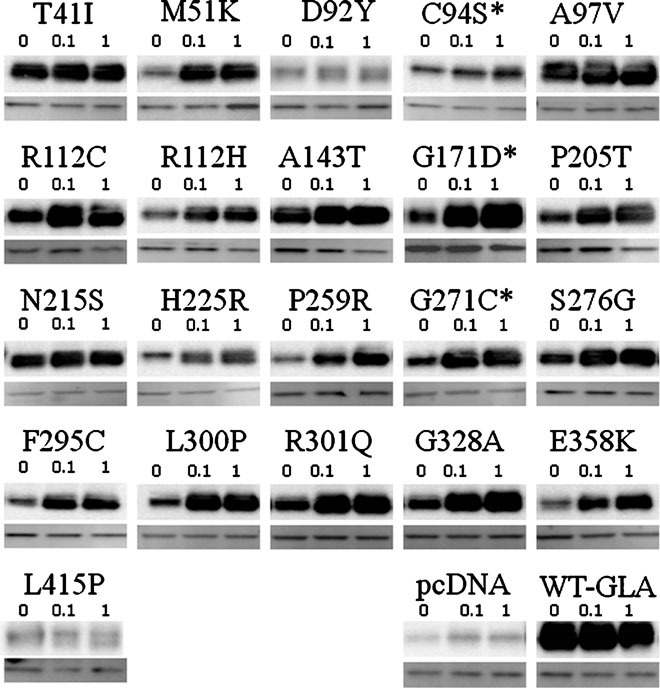
Western blot results for representative mutant forms of α-Gal A. HEK-293 cells transiently transfected with the indicated mutant forms of α-Gal A were incubated for 4 to 5 days without or with 0.1 or 1 mM AT1001 (represented by the numbers above the bands). Cell lysates were prepared as described for the enzyme assay and total protein concentrations were measured. An equal amount of total protein (1 µg) was loaded into each lane and Western blotting was performed according to standard protocols (see Materials and Methods). Mutant forms that showed a positive response to AT1001 in the α-Gal A activity assay generally showed increases in α-Gal A protein levels after incubation with AT1001. Those showing an increase in the protein but no increase in the enzyme activity assay after incubation with AT1001 are indicated by an asterisk (*). Western blots of GAPDH as an additional control for equal protein loading in each lane are also provided. Representative blots of pcDNA only and wild-type *GLA* transfected HEK-293 cells are provided as negative and positive controls. The data for the controls and for each mutant form are representative of at least three independent experiments with similar results.

To determine if the increases in α-Gal A activity seen in the HEK-293 cell lysates were accompanied by increases in cellular levels of the enzyme, α-Gal A protein levels were measured directly by Western blotting for a subset of mutant forms. The Western blot results were generally consistent with the enzyme assay results. As shown in Figure 3, mutant forms that were increased by AT1001 in the enzyme assay consistently showed robust increases in α-Gal A protein levels (e.g., R301Q [c.902G>A] and N215S [c.644A>G]). Similarly, mutant forms that were not increased by AT1001 in the enzyme assay generally showed little to no increase in protein level by Western blot (D92Y [c.274G>T] and L415P [c.1244T>C]). However, for some mutant forms such as C94S (c.281G>C), G171D (c.512G>A), and G271C (c.811G>T), activity was not increased by AT1001 in the enzyme assay, but robust increases in protein levels were observed. Although these mutant forms are likely to be catalytically inactive as indicated by a lack of baseline enzyme activity (Table 1) and as suggested by amino acid substitution within or near critical α-Gal A structural determinants [Garman and Garboczi, 2004], the increase in protein levels shown by Western blot suggests that they retain the ability to bind to AT1001.

### Locations of Point Mutations in Mutant Forms of α-Gal A

**Figure 4:**
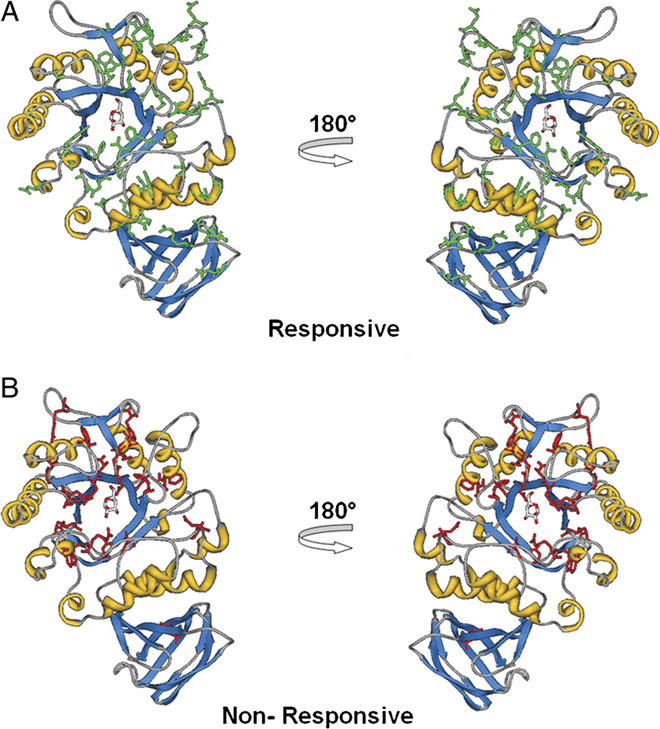
Locations of residues with point mutations in mutant forms of α-Gal A. Eighty-one amino acid residues corresponding to the missense mutations tested in the HEK-293 cell-based assay were mapped onto the structure of the α-Gal A monomer. The α-Gal A monomeric structure is shown in ribbon representation with the bound galactose ligand and affected residues displayed in stick format. Residues with point mutations in AT1001-responsive (**A**) and nonresponsive (**B**) mutant forms of α-Gal A are colored green and red, respectively.

The locations of the residues affected by single missense mutations were highlighted in the monomeric α-Gal A protein structure to assess if structural determinants can be identified for response to AT1001 (Fig. 4) [Garman and Garboczi, 2004]. Residues mutated in responsive mutant forms of α-Gal A were found throughout the (β/α)_8_ domain and the C-terminal antiparallel β-sheet domains, indicating that they occur throughout the full-length protein (Fig. 4A). In contrast, many residues that are mutated in nonresponsive forms of α-Gal A were located within, or in close proximity to, the active site (Fig. 4B). However, several other residues mutated in nonresponsive forms were located elsewhere throughout the full-length protein without a straightforward structural relationship. Interestingly, mutation at some sites, such as F113 generated both responsive (F113L, c.337T>C) and nonresponsive mutant forms (F113S, c.338T>C). These observations further support the need for empirical testing of each Fabry disease-causing mutant form of α-Gal A to identify those that are responsive to AT1001.

### Comparison of the Mutant α-Gal A Responses Observed in HEK-293 Cells and in Fabry Patient Lymphoblasts

**Figure 5:**
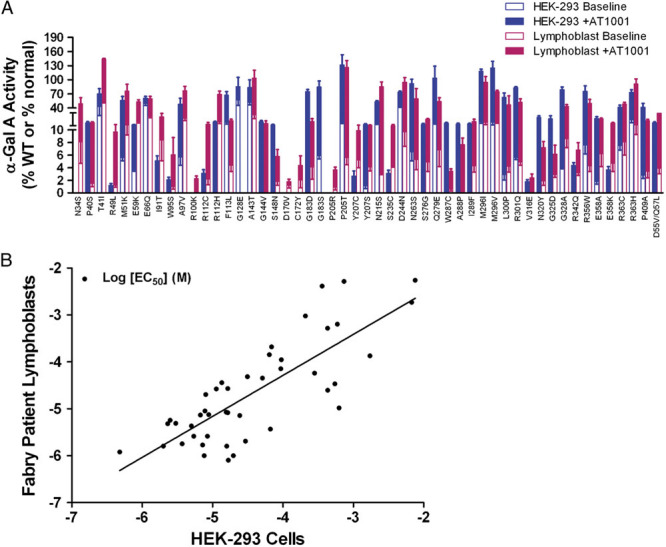
The mutant α-Gal A responses to AT1001 in HEK-293 cells are comparable to those from Fabry patient lymphoblasts. **A:** The HEK-293 cell and Fabry patient lymphoblast [Benjamin et al., 2009] average baseline and maximally increased α-Gal A activity after AT1001 incubation are shown for 51 different mutant forms that were responsive in either assay (the data are not shown for 24 other mutant forms that consistently did not respond to AT1001 in both assays). The HEK-293 cell data have been normalized to the baseline α-Gal A activity in wild-type-transfected HEK-293 cells. The patient lymphoblast data have been normalized to the baseline α-Gal A activity in normal human lymphoblasts. **B:** Correlation analysis of the average EC_50_ values (expressed as the logEC_50_ [M]) for AT1001-increased α-Gal A activity of 44 different mutant forms that were responsive in both assays is shown. The logEC_50_ values of the AT1001-mediated responses were significantly correlated (Pearson correlation coefficient [*r*] was 0.793 with a two-tailed *P*-value of <0.0001) between the HEK-293 cell and the patient lymphoblast assays. Significant correlations of the baseline α-Gal A activity and the maximum α-Gal A activity after AT1001 incubation were also found between these two assays (Pearson correlation coefficients [*r*] were 0.760 and 0.751, respectively, with two-tailed *P*-values <0.0001 and *n* = 75 for each; plots not shown).

Previous studies have shown that AT1001 increases the cellular levels and activity of some missense mutant forms of α-Gal A in lymphoblasts derived from Fabry patients [Benjamin et al., 2009; Fan et al., 1999a; Ishii et al., 2007; Shin et al., 2007]. To assess if the concentration-dependent, AT1001-mediated increases in α-Gal A activity seen in the HEK-293 cell-based assay are consistent with those published previously in male Fabry patient lymphoblasts [Benjamin et al., 2009], the responses of 75 different mutant forms that have been tested in both cell types were compared. As shown in Figure 5A, 44 of 75 mutant forms consistently showed a significant response to AT1001 in both cell types. Twenty-four mutant forms consistently did not respond to AT1001 in either cell type. Seven mutant forms showed inconsistent results in the two assays (Fig. 5A). Five of these, N34S (c.101A>G), R100K (c.299G>A), D170V (c.509A>T), C172Y (c.515G>A), and P205R (c.614C>G) were responsive in patient-derived lymphoblasts only, but with low baseline activity (generally <1% of wild type) and small increases in α-Gal A levels (below 3% of wild type), or relatively high EC_50_ values (>300 µM) [Benjamin et al., 2009]. Two (G128E [c.383G>A] and G183S [c.547G>A]) were responsive in the HEK-293 cell-based assay, but not in the patient lymphoblasts (see Discussion).

The degree of consistency between these two sets of results for these 75 mutant forms was evaluated in two ways: first by correlation analysis, and second by calculating the sensitivity and specificity [Altman and Bland, 1994a,b] of the comparison. Correlation analysis showed that the baseline α-Gal A activity, the maximum α-Gal A activity after AT1001 incubation, and the potency (Fig. 5B) of the AT1001-mediated response were significantly correlated (Pearson correlation coefficients [*r*] were 0.760 [*n* = 75], 0.751 [*n* = 75], and 0.793 [*n* = 44], respectively, with two-tailed *P*-values <0.0001 for each) between the HEK-293 cell and the patient lymphoblast assays. The sensitivity of the HEK-293 cell results in this comparison to male Fabry patient lymphoblast results was calculated to be 0.90 (44÷49 = 0.90). The specificity of the HEK-293 cell results in this comparison to the male Fabry patient lymphoblast results was calculated to be 0.92 (24÷26 = 0.92). Collectively, these results indicate a high degree of consistency in the responsiveness of α-Gal A mutant forms as shown in the HEK-293 cell-based assay and in male Fabry patient lymphoblasts.

### Comparison of the Mutant α-Gal A Responses Observed in HEK-293 Cells and in PBMCs of Fabry Patients Treated with AT1001

To determine if the HEK-293 cell-based assay can be used to identify mutant forms of α-Gal A that are most likely to respond to AT1001 in vivo, the degree of consistency between the HEK-293 cell-based assay results and the observed responses of mutant α-Gal A in PBMCs of male Fabry patients after oral administration of AT1001 was evaluated. To this end, the HEK-293 cell α-Gal A activity at the clinically achievable concentration of 10 µM AT1001 [Schiffmann et al., 2008] was determined (see Materials and Methods) for the 19 mutant forms of α-Gal A represented in the *FAB-CL-201, FAB-CL-202*, and *FAB-CL-203* clinical studies. Then, the α-Gal A responses from HEK-293 cells were compared to the patient PBMC α-Gal A responses from the Phase 2 studies (Table 2). The degree of consistency between these two sets of results was first evaluated by calculating the sensitivity and specificity of the HEK-293 cell-based results in this comparison for these 19 mutant forms. Second, the degree of consistency was evaluated by correlation analysis.

**Table 2:** Comparison of the Mutant α-Gal A Responses Observed in HEK-293 Cells and in PBMCs of Fabry Patients Treated with AT1001

	Genotype/subject			HEK-293 cell-based assay			Phase 2 PBMC Assay					
			
Source	Protein change	cDNA change	Subject	−AT1001 (% WT)	+AT1001 (% WT)	Responsive at 10 µM AT1001?	−AT1001 (% normal)	+ AT1001 (% normal)				PBMC response
**FAB-CL-203&FAB-CL-202**								**Wk 4**	**Wk 12**	**Wk 24**	**Wk 48**	
								150 mg	150 mg	150 mg	150 mg	
								qod	qod	qod	qod	
	**p.C94S**	**c.281G>C**	**1**	**0**	**0**	**No**	**0**	**0**	**0**	**1**	**0**	**Non/limited**
	**p.R112C**	**c.334C>T**	**2**	**0**	**0**	**No**	**0**	**2**	**0**	**1**	**2**	**Non/limited**
	p.P205T	c.613C>A	3	18.2	62.5	Yes	1	2	2	6	14	Good
	p.N215S	c.644A>G	4	15.7	40.7	Yes	16	38	48	50	34	Good
	*p.F295C*	*c.884T>G*	*5*	*4.1*	*12.3*	*Yes*	*0*	*1*	*1*	*2*	*1*	*Non/limited*
	p.P259R	c.776C>G	6	28.1	67	Yes	1	10	10	ND	2	Good
			7				1	13	11	15	NA	Good
	p.R301Q	c.902G>A	8	5.6	49.5	Yes	1	34	33	32	28	Good
	**p.L415P**	**c.1244T>C**	**9**	**0**	**0**	**No**	**1**	**1**	**0**	**1**	**1**	**Non/limited**
**FAB-CL-201**								**Wk 2**	**Wk 4**	**Wk 6**	**Wk 12**	
								25 mg	100 mg	250 mg	50 mg	
								bid	bid	bid	qd	
	p.T41I	c.122C>T	10	43.1	58.4	Yes	21	89	122	127	112	Good
			11				30	74	122	151	112	Good
	p.M51K	c.152T>A	12	5.6	25.1	Yes	4	24	30	20	18	Good
	p.A97V	c.290C>T	13	5.7	31.6	Yes	4	53	66	70	67	Good
	p.A143T	c.427G>A	14	52	69.4	Yes	24	66	60	64	79	Good
	p.S276G	c.826A>G	15	0	3.7	Yes	0	4	8	6	4	Good
	p.L300P	c.899T>C	16	2.9	25.6	Yes	1	7	7	13	2	Good
	p.G328A	c.983G>C	17	3.9	22.1	Yes	0	2	2	6	2	Good
**FAB-CL-201***in vivo***screen**								**Wk 2**	–	–	–	
								150 mg				
								qd				
	**p.G171D**	**c.512G>A**	**18**	**0**	**0**	**No**	**2**	**2**	**ND**	**ND**	**ND**	**Non/limited**
	**p.H225R**	**c.674A>G**	**19**	**0**	**0**	**No**	**2**	**1**	**ND**	**ND**	**ND**	**Non/limited**
	**p.G271C**	**c.811G>T**	**20**	**0**	**0**	**No**	**27**	**0**	**ND**	**ND**	**ND**	**Non/limited**
	p.R301Q	c.902G>A	21	5.6	49.5	Yes	2	12	ND	ND	ND	Good
	**p.R227Q**	**c.680G>A**	**22**	**0**	**0**	**No**	**1**	**0**	**ND**	**ND**	**ND**	**Non/limited**

The HEK-293 cell-based assay results were compared to the α-Gal A responses in PBMCs of male subjects in Phase 2 studies (see also ClinicalTrials.gov). Subjects enrolled in FAB-CL-201 (NCT00214500) were orally administered AT1001 at doses of 25 mg twice a day for the first 2 weeks, 100 mg twice a day during weeks 3 and 4, 250 mg twice a day during weeks 5 and 6, followed by 50 mg once per day during weeks 7 through 12. Separately, five other subjects (indicated by the “FAB-CL-201 in vivo screen” section) were orally administered 150 mg AT1001 every day for 2 weeks during a screening period and then tested for an in vivo PBMC α-Gal A response (this in vivo screen was conducted under a protocol amendment). None of these subjects, who met the other eligibility criteria (see ClinicalTrials.gov), responded with increased PBMC α-Gal A levels after AT1001 administration, and thus did not participate in the remainder of the study. Subjects enrolled in FAB-CL-202 (NCT00283959) and FAB-CL-203 (NCT00283933) were orally administered 150 mg AT1001 every other day for the duration of the weeks indicated. In the PBMC assay, the baseline α-Gal A activity (−AT1001) presents the values from day 1, the last predose sample. In the PBMC assay, the α-Gal A activity (+AT1001) presents the values after 150 mg (FAB-CL-202, FAB-CL-203, and FAB-CL-201 in vivo screen) or 25, 50, 100, or 250 mg (FAB-CL-201) AT1001 administration at different regimens (“qd,” “qod,” and “bid” refer to “every day,” “once every other day,” and “twice per day”) and time points (“Wk” refers to “week”) as specified in accordance with the different clinical protocols. Subjects were categorized according to their maximal net α-Gal A increase from baseline after treatment with AT1001. Subjects with a 3% of normal or greater net increase were categorized as “good responders,” and subjects with less than a 3% of normal net increase were categorized as “non/limited responders.” The mean normal α-Gal A activity in PBMCs from healthy volunteers was 22 nmol of free 4-MU released/mg protein/hr. “ND” refers to “not determined.” “NA” refers to “not available” due to insufficient total protein concentration (below the limit of quantification) in the sample. In the HEK-293 cell-based assay, mutant forms that were considered responsive for this comparison had statistically significantly greater α-Gal A activity at 10 µM AT1001 compared to baseline. Black text indicates that the mutant form of α-Gal A showed a response in both assays, bold text indicates that the mutant form of α-Gal A did not show a response in either assay, and italic text indicates that the mutant form of α-Gal A was responsive only in the HEK-293 cell-based assay.

Fourteen male Fabry patients representing 11 different mutant forms showed positive PBMC α-Gal A responses to oral administration of AT1001 (defined as an increase in α-Gal A levels of at least 3% of normal; also referred to as a “good” α-Gal A response) (Table 2). Furthermore, 3 of these 11 mutant forms showed in vivo responses to AT1001 in two different patients. Those same 11 mutant forms consistently had responses to 10 µM AT1001 that were statistically significant in the HEK-293 cell-based assay. Thus, the sensitivity of the HEK-293 cell-based assay results in comparison to male patient PBMC α-Gal A responses for this small number of mutant forms shared between the two datasets was calculated to be 1.0 (11÷11 = 1.0).

Eight male Fabry patients representing eight different mutant forms showed “non/limited” PBMC α-Gal A responses to oral administration of AT1001 (defined as an increase in α-Gal A levels of less than 3% of normal) (Table 2). Consistently, 7 of those 8 mutant forms of α-Gal A did not have a statistically significant increase in α-Gal A activity after incubation with 10 µM AT1001 in the HEK-293 cell-based assay. However, the results were inconsistent for one mutant form of α-Gal A, F295C (c.884T>G). F295C did not show a “good” response in PBMCs from a patient with this mutation when he was receiving a dose of 150 mg AT1001 every other day. In contrast, this mutant form did show a statistically significant response to 10 µM AT1001 in the HEK-293 cell-based assay. Thus, the specificity of the HEK-293 cell-based assay results in comparison to male patient PBMC α-Gal A responses for this small number of mutant forms shared between the two datasets was calculated to be 0.88 (7÷8 = 0.88).

The consistency of the HEK-293 cell-based and in vivo responses was further evaluated by correlation analysis. This analysis showed that the baseline α-Gal A activity and the α-Gal A activity after treatment with AT1001 (i.e., after AT1001 oral administration to patients in vivo versus after 10 µM AT1001 incubation in vitro) were significantly correlated (Pearson correlation coefficients [*r*] were 0.639 and 0.559, respectively, with two-tailed *P*-values <0.01 and *n* = 22 for each) between the HEK-293 cell and the PBMC assays. Collectively, these results indicate a high degree of consistency in the responses of this small subset of α-Gal A mutant forms as assessed in the in vitro HEK-293 cell-based assay and in male patient PBMC α-Gal A responses after oral administration of AT1001.

## Discussion

AT1001 (migalastat hydrochloride, 1-deoxygalactonojirimycin) is a pharmacological chaperone for α-Gal A that is in Phase 3 clinical development as a potential therapy for Fabry disease. AT1001 selectively binds and stabilizes α-Gal A, increasing total cellular levels and activity for some mutant forms (defined as “responsive”). In general, missense mutations in *GLA* often lead to the expression of responsive mutant forms of α-Gal A and constitute approximately 60% of all known Fabry disease-causing mutations (more than 600 have been identified to date) [Benjamin et al., 2009; Fan et al., 1999a; Ishii et al., 1996, 2007; Lemansky et al., 1987; Shin et al., 2008; Yam et al., 2005, 2006]. In the current study, we developed a cell-based assay in HEK-293 cells to identify missense mutant forms of α-Gal A that are responsive to AT1001. Furthermore, we demonstrated that the responses seen in this assay can identify mutant forms of α-Gal A that respond to AT1001 inpatient-derived cell lines and in PBMCs of Fabry patients administered the drug.

The assay was designed to test the ability of AT1001 to increase the total cellular activity of different mutant forms of α-Gal A following expression in HEK-293 cells. The specific effect of AT1001 on the expressed mutant form of α-Gal A was determined by subtraction of the endogenous HEK-293 cell wild-type α-Gal A activity as a function of AT1001 concentration. Thus, increased mutant α-Gal A activity in HEK-293 cell lysates after incubation with AT1001 indicated increased total cellular levels of the α-Gal A mutant form. We used this approach to test 81 different Fabry disease-associated missense mutant forms that were represented in our collection of male Fabry patient-derived lymphoblasts and in male patients enrolled in Phase 2 clinical trials of AT1001. AT1001 mediated significant increases in the α-Gal A levels of approximately 60% (49 of the total 81) of the mutant forms. These mutant forms represented a wide range of baseline α-Gal A levels, and responded with a range of relative increases in α-Gal A levels, and EC_50_ values, consistent with results from Fabry patient cell lines [Benjamin et al., 2009; Shin et al., 2007, 2008]. α-Gal A protein structural mapping of the amino acid residues affected by the 81 missense mutations did not identify obvious structural determinants that could be used to consistently predict the mutant α-Gal A response to AT1001 [Garman, 2007; Garman and Garboczi, 2004; Matsuzawa et al., 2005; Shabbeer et al., 2006]. Collectively, these results further support the need for empirical testing of each Fabry disease-associated mutant form to identify those that are responsive to AT1001 [Benjamin et al., 2009; Shin et al., 2007, 2008].

For the subset of mutant forms that were also tested by Western blot, those that showed increased α-Gal A activity after incubation with AT1001 also consistently showed increased α-Gal A protein levels. Interestingly, the Western blot results revealed increased protein levels for some mutant forms that did not show an increase in activity (e.g., C94S). These mutant forms also did not have measurable baseline activity, suggesting little to no catalytic activity; however, we cannot rule out the possibility that these can metabolize the natural substrate, GL-3 in the natural cellular environment. These results suggest that AT1001 may have the ability to bind, stabilize, and promote lysosomal trafficking of some catalytically *incompetent* mutant forms. If mutant α-Gal A accumulation in the ER leads to cellular stress and contributes further to cellular dysfunction and disease [Ishii et al., 1996], it is interesting to speculate whether this may be another mechanism through which AT1001 could have therapeutic benefit. This may warrant further study.

The responses of 75 different mutant forms of α-Gal A to AT1001 that were seen in the HEK-293 cell-based assay were generally consistent with those observed in male Fabry patient lymphoblast cell lines [Benjamin et al., 2009]. In this comparison, 90% of the mutant forms that were responsive in male Fabry patient lymphoblasts were also responsive in HEK-293 cells, whereas 92% of those that were nonresponsive in male Fabry patient lymphoblasts were also not responsive in HEK-293 cells. Furthermore, baseline and maximum α-Gal A activity, as well as the potency of the AT1001-mediated response were significantly correlated between the two in vitro assays. This further indicates that the response to AT1001 predominantly depends on the mutant form of α-Gal A that is expressed, and in general, is not dependent on differences in cell type, expression level, or source of the gene [Asano et al., 2000; Benjamin et al., 2009; Fan et al., 1999b; Ishii et al., 2007; Shin et al., 2007, 2008]. However, the responses to AT1001 did differ for seven of the mutant forms. In lymphoblasts, five of these mutants (N34S [c.101A>G], R100K [c.299G>A], D170V [c.509A>T], C172Y [c.515G>A], and P205R [c.614C>G]) showed low baseline activity (generally <1% of wild-type) and small increases in α-Gal A levels (below 3% of wild type), or relatively high EC_50_ values (>300 µM) [Benjamin et al., 2009]. In HEK-293 cells, no enzyme activity was quantifiable at baseline or after AT1001 incubation for these mutant forms. Thus, although the response outcomes were somewhat different, importantly, both assays did consistently show low enzyme activity and minimal or no response to AT1001 for these mutant forms. The differences in response that were seen may have resulted from subtle assay-related differences (e.g., cellular background, gene source, expression level, or assay sensitivity at the limit of quantification), suggesting that these mutant forms are severely impaired with a very limited capacity for pharmacological chaperone rescue. On the other hand, two mutant forms (G128E [c.383G>A] and G183S [c.547G>A]) showed responses only in the HEK-293 cells, but not in lymphoblasts. The difference in response of G183S is likely due to the underlying nucleotide change at the genomic level that leads to a splicing aberration [Lai et al., 2003], resulting in a severely altered α-Gal A protein sequence in patient lymphoblasts. The nucleotide change at the cDNA level, however, leads to a straightforward single amino acid residue change in the α-Gal A protein sequence, which is the mutant form tested in the HEK-293 cell-based assay. The reason for the difference in the G128E response to AT1001 is less clear. G128E was not responsive in the lymphoblasts, consistent with results from other male patient cell lines with the same mutation [Shin et al., 2008]. It is possible that this mutation at the genomic level may also lead to additional defects, such as aberrant splicing or instability of the mRNA transcript.

It should also be noted that every normal lymphoblast line tested showed a concentration-dependent increase in wild-type α-Gal A levels in response to AT1001 [Benjamin et al., 2009]. Similarly, endogenous α-Gal A in HEK-293 cells is responsive to AT1001 (as measured by enzyme activity and Western blot; data not shown), and therefore its activity as a function of AT1001 concentration must be subtracted from that of each mutant form in the HEK-293 cell-based assay. In contrast, transiently transfected wild-type α-Gal A resulted in very high baseline levels of activity that did not consistently show a concentration-dependent increase after incubation with AT1001. This effect may be due to high-level expression of α-Gal A by the transfected HEK-293 cells that leads to saturation of the cellular machinery that regulates folding and/or trafficking of the enzyme, thus preventing any further increase with AT1001.

Most importantly, the responses to AT1001 of 19 different α-Gal A mutant forms in the HEK-293 cell-based assay were generally consistent with observed increases in α-Gal A activity in PBMCs from male Fabry patients orally administered AT1001 during Phase 2 clinical studies. In this comparison, every mutant form of α-Gal A that did respond in Fabry patient PBMCs also showed a response to a clinically achievable concentration of AT1001 (10 µM) in the HEK-293 cell-based assay. Eighty-eight percent of those that did not respond in Fabry patient PBMCs also did not respond to AT1001 in the HEK-293 cell-based assay. Furthermore, the baseline α-Gal A activity and the α-Gal A activity after treatment with AT1001 were significantly correlated between the HEK-293 cell-based and the in vivo assays. These observations suggest that the mutant α-Gal A responses to a clinically achievable concentration of AT1001 in the HEK-293 cell-based assay can identify mutant forms of α-Gal A that are likely to respond to AT1001 in vivo.

The degree of consistency found in the current comparison of HEK-293 cell-based and in vivo mutant α-Gal A responses does not necessarily indicate the degree of consistency that would be seen with any particular dose or regimen of AT1001. This is because the in vivo α-Gal A responses analyzed here were obtained following the administration of different AT1001 doses (50, 150, or 250 mg) and with various regimens (every other day, twice per day, or every day), according to the different clinical study protocols. Although it is possible that some mutant forms might show a limited response at a lower dose and a larger response at a higher dose, our results indicate that most mutant forms that were tested at both lower and higher doses showed a “good” response at either dose. This suggests that a single common dose and regimen of AT1001 may be sufficient to mediate a response of the enzyme in vivo for many mutant forms of α-Gal A.

Fabry patients with responsive mutant forms of α-Gal A potentially are most likely to benefit from AT1001 treatment. Thus, several approaches have been considered to determine if a candidate patient's disease-associated mutant form of α-Gal A is likely to be responsive to AT1001. These involve measurement of the residual α-Gal A activity (e.g., in PBMCs or plasma), the α-Gal A response to AT1001 in vivo or ex vivo in primary cultured T cells, and/or the urine GL-3 response to AT1001 following oral administration [Benjamin et al., 2009; Desnick et al., 2001; Ishii et al., 2007]. These approaches may be limited by a lack of consistency with the in vivo response of the patient's α-Gal A mutant form, or exposure to AT1001 before the potential for therapeutic benefit is known. Most importantly, these approaches do not provide meaningful results for many female Fabry patients. As Fabry disease is X-linked, female patients are mosaic, harboring a mixture of cells that express either wild-type or mutant α-Gal A. In samples derived from female patients, the measured α-Gal A activity is dominated by the wild-type enzyme, which is responsive to AT1001; hence, neither the baseline activity nor the effect of AT1001 on the mutant form can be accurately determined. Importantly, in contrast to female patient cell lines or samples, the α-Gal A responses determined in the HEK-293 cell-based assay are purely the response of the heterologously-expressed mutant form.

Male Fabry patients express one mutant copy of the *GLA* gene in all cells. Thus, α-Gal A responses to AT1001 in males could be determined in vivo or in cultured cells and used to select female patients with the same mutation; however, the α-Gal A response to AT1001 from male patients or male cell lines is available for only a limited number of mutant forms [Benjamin et al., 2009]. Recently, a computational approach to predicting the mutant α-Gal A response based on protein sequence has been proposed and could be applicable for selection of female Fabry patients; but comparison to responses measured in male patient cell lines showed relatively low positive predictive value and sensitivity (0.66 and 0.49, respectively), whereas a comparison to in vivo responses was not available [Andreotti et al., 2010]. Although the more conventional measurement of urine GL-3 has also been considered, baseline substrate levels are often within the normal range in females [Kitagawa et al., 2005, 2008; Vedder et al., 2008], thereby making it difficult to measure the effect of AT1001 based on this endpoint. Thus, the selection of female Fabry patients continues to warrant a test for the AT1001 response of the α-Gal A mutant form. As such, the HEK-293 cell-based assay may prove to be a useful complementary approach.

The degree of consistency between the HEK-293 cell-based and in vivo responses of α-Gal A mutant forms should continue to be assessed in future studies that include a larger number of mutant forms. Preferably, the clinical studies would assess the effect of a fixed dose and regimen of AT1001 on α-Gal A levels in vivo, and would also include different patients that harbor the same mutation to assess the consistency of the intersubject mutant α-Gal A response. Furthermore, criteria that define the threshold response to a clinically achievable concentration in the HEK-293 cell-based assay should be refined to better identify those mutant forms that are most likely to respond to AT1001 in vivo. It may be challenging to obtain the highest degree of consistency between the HEK-293 cell-based and in vivo results for mutant forms with responses to AT1001 that are more dependent on other factors such as different cell or tissue types, mutant α-Gal A expression levels, or subtle differences in the target tissue concentration of AT1001. Furthermore, the relationship between the in vivo α-Gal A response and clinical efficacy remains to be determined. Although increases of 1 to 5% of normal enzyme activity in vivo have been suggested to be clinically meaningful [Conzelmann and Sandhoff, 1983; Desnick, 2004], the threshold level of increase that will lead to clinical efficacy is currently unknown. In addition, clinical efficacy can be affected by other factors, such as disease severity, extent of disease progression at the start of treatment, individual differences in AT1001 exposure levels at a given dose, and/or genetic modifiers.

In conclusion, a high degree of consistency in the responses to AT1001 of α-Gal A mutant forms determined in the HEK-293 cell-based assay was shown in comparison to the in vivo responses assessed in PBMCs of male Fabry patients orally administered AT1001 during Phase 2 clinical trials. This indicates that the responses to a clinically achievable concentration of AT1001 in the HEK-293 cell-based assay can identify mutant forms of α-Gal A that are likely to respond to AT1001 in vivo. Thus, the HEK-293 cell-based assay may prove to be a useful complementary aid in the identification of Fabry patients with AT1001-responsive mutant forms. Further evaluation of the utility of the HEK-293 cell-based assay for Fabry patient selection for treatment with AT1001 is ongoing.
